# New insights into the effects of irisin levels in HIV-infected subjects: correlation with adiposity, fat-free mass, and strength parameters

**DOI:** 10.1590/2359-3997000000270

**Published:** 2017-06-14

**Authors:** Joice Cristina dos Santos Trombeta, Jonato Prestes, Dahan da Cunha Nascimento, Ramires Alsamir Tibana, Guilherme Borges Pereira, Thiago da Rosa Lima, Géssica Alves Fraga, Roberto Carlos Vieira-Junior, Fabrício Azevedo Voltarelli

**Affiliations:** 1 Universidade Estadual de Campinas Campinas SP Brasil Universidade Estadual de Campinas (Unicamp), Campinas, SP, Brasil; 2 Universidade Federal de Mato Grosso Cuiaba MT Brasil Universidade Federal de Mato Grosso (UFMT), Cuiaba, MT, Brasil; 3 Universidade Católica de Brasília Brasília DF Brasil Programa de Pós-Graduação em Educação Física, Universidade Católica de Brasília (UCB), Brasília, DF. Brasil; 4 Universidade do Estado de Mato Grosso Caceres MT Brasil Universidade do Estado de Mato Grosso (Unemat), Caceres, MT, Brasil

**Keywords:** Body composition, HIV infection, irisin levels, muscle strength

## Abstract

**Objective:**

Patients infected with the human immunodeficiency virus (HIV) have an increased risk of metabolic disorders and alterations on irisin levels. Therefore, the purpose of the current investigation was to quantify the circulating irisin concentration in HIV-infected subjects under highly active antiretroviral therapy and to determine possible correlations between irisin levels with fat mass, fat-free mass, body mass index (BMI), and muscle strength.

**Subjects and methods:**

Cross-sectional study of 10 men (36.7 ± 11.3 years) and 10 women (42.5 ± 10.3 years) infected with HIV, recruited from the Specialized Service Center in the State Center of Reference for High and Medium Complexity. Blood samples were collected to determine plasma irisin levels, glucose, HDL, total cholesterol, triglycerides, and LDL. Body composition (fat mass, fat-free mass) and anthropometrics (body mass index; BMI) were measured by bioelectrical impedance. Muscle strength was assessed using a mechanic hand dynamometer and one maximum repetition tests.

**Results:**

Irisin levels correlated positively with fat mass (r = 0.67; p = 0.001) and BMI (r = 0.48; p = 0.036). In contrast, there was an inverse correlation between irisin levels and fat-free mass (r = -0.41; p = 0.008) and five strength parameters: right hand grip (r = -0.46; p = 0.044); left hand grip (r = -0.50; p = 0.027), relative hand grip (r = -0.79; p = 0.001), bench press (r = -0.58; p = 0.009), leg press (r = -0.40; p = 0.085), and biceps curl (r = -0.059; p = 0.009).

**Conclusion:**

Irisin levels correlated positively with body fat and negatively with fat-free mass and strength parameters in HIV-infected patients. Female patients infected with HIV receiving highly active antiretroviral therapy have higher levels of irisin compared with men in a similar circumstance.

## INTRODUCTION

The skeletal muscle is recognized as an endocrine organ expressing and releasing myokines with autocrine and paracrine effects (1). The peroxisome proliferator-activated receptor γ coactivator 1-α (PGC1-α) is an important molecule expressed in the muscle that plays many biological functions related to energy metabolism (2). Muscle production of PGC1-α is stimulated by exercise and potentially induces the expression and secretion of several muscle gene products such as fibronectin type III domain containing 5 (FNDC5). The *FNDC5* gene is processed proteolytically to form irisin, which in turn induces profound changes in subcutaneous adipose tissue by stimulating brown adipose tissue and *uncoupling protein 1* (*UCP1*) expression. Irisin may be a new target for the treatment of metabolic disorders based on its potential to improve the metabolic profile and increase whole body energy expenditure (2).

The skeletal muscle is the main source of circulating irisin in animal models and humans (3,4). Rats infected with the human immunodeficiency virus (HIV) lose weight (predominantly due to muscle wasting, with minor changes in fat proportion) (5), which could in part decrease irisin secretion. In a subcohort of morbidly obese subjects with metabolic disorders, circulating irisin levels were significantly associated with *FNDC5* gene expression in subcutaneous and visceral adipose tissues instead of muscle mass (6,7).

Abnormalities in fat distribution have been described as the most prevalent adverse effect of highly active antiretroviral therapy (HAART) in individuals with HIV (8). A working hypothesis for the responsible mechanisms implicates mitochondrial bioenergetics disturbances characterized by mitochondrial toxicity. Mitochondrial toxicity leads to the activation of lipolysis, apoptosis, and abnormal release of proinflammatory cytokines (TNF-alpha, IL-6). This cascade of events may be responsible for the subsequent alteration in the overall metabolism, characterized by insulin resistance, dyslipidemia, and vascular damage, creating an imbalance of the anabolic and catabolic systems and resulting in loss of muscle mass and function (9,10).

Further evidence suggests that irisin levels are associated with body mass index (BMI). Stengel and cols. verified that under pathological conditions, obese individuals with BMI ≥ 40 kg/m^2^ and mean age of 48 years exhibited higher circulating irisin levels when compared with individuals characterized as having a normal weight and being anorexic (11). Furthermore, plasma irisin levels correlated positively with body mass, fat mass, BMI, fat-free mass, waist-to-hip ratio, and insulin levels. The authors speculated that the higher irisin levels found in obese individuals might also represent a physiological attempt to restore glucose tolerance. In addition, irisin correlated positively with fasting insulin, homeostasis model assessment of insulin resistance (HOMA-IR), age, cholesterol ratio, and BMI in patients with gestational diabetes (12). Furthermore, irisin levels are elevated in individuals with HIV compared with those without HIV and are unaffected by lifestyle modifications, including exercise (13). Therefore, the available data regarding irisin and body composition in humans are inconclusive, especially in HIV-infected subjects.

Thus, the aim of the present study was to quantify the circulating irisin concentration in individuals with HIV undergoing HAART and identify potential correlations between irisin levels and fat mass, fat-free mass, BMI, and muscle strength. The initial hypothesis was that irisin levels would correlate positively with fat mass and negatively with fat-free mass and strength parameters in this population.

## SUBJECTS AND METHODS

### Participants

Twenty volunteers with HIV undergoing HAART were recruited from a Specialized Service Center in the State Center of Reference for High and Medium Complexity (Cuiaba-MT, Brazil). Ten participants were men (36.7 ± 11.3 years), and ten were women (42.5 ± 10.3 years). The participants were older than 18 years, were infected with HIV, and were receiving HAART for ≥ 12 weeks, with a cumulative HAART duration of 6.40 ± 5.93 years. Eligible participants were adults classified as “sedentary” by the International Physical Activity Questionnaire (14,15) and without contraindications for exercise identified during a cardiovascular exercise stress test. All participants were under antiretroviral therapy. The HAART contained a combination of nucleoside-analogue reverse transcriptase inhibitors (NRTIs), non-nucleoside reverse transcriptase inhibitors (NNRTIs), protease inhibitors (PIs), and integrase inhibitors (IIs). Sixteen participants were receiving two types of NRTIs and one type of NNRTI; two participants were receiving two PIs and three NRTIs; one participant was receiving two PIs and two NRTIs; and one participant was receiving two PIs, two NRTIs, and one II. None of the participants showed a positive diagnosis for lipodystrophy syndrome.

Potential participants were excluded if presenting an acute or chronic inflammatory condition (besides HIV), pregnant/lactating, having prior myocardial infarction and vascular diseases, receiving systemic chemotherapy or steroids, having uncontrolled diabetes mellitus, using anabolic agents and growth hormone, or showing a CD4+ T cell count below 200 cells/mm^3^. Table 1 shows the characteristics of the overall cohort. The study design and employed procedures are in accordance with ethical standards and the Declaration of Helsinki. All subjects were fully informed about the risks and benefits associated with the participation in the study and gave their written informed consent for participation. The study was approved by the Research Ethics Committee at Julio Muller University Hospital (N: 673/09).

**Table 1:** Baseline characteristics of the participants presented as mean ± standard deviation (SD)

	Mean ± SD in all subjects	Mean ± SD in men (n = 10)	Mean ± SD in women (n = 10)
**Anthropometric measures**			
Body weight, kg	70.64 ± 17.88	75.47 ± 12.74	65.91 ± 21.51
Height, cm	1.64 ± 0.10	1.70 ± 0.08	1.57 ± 0.08
BMI, kg/m^2^	26.53 ± 8.01	26.03 ± 4.83	27.03 ± 10.57
Fat mass, %	30.57 ± 11.29	29.95 ± 8.52	34.19 ± 12.94
Fat mass, kg	23.00 ± 13.88	21.06 ± 10.22	24.95 ± 17.15
Fat-free mass, %	69.47 ± 11.36	73.03 ± 8.57	65.92 ± 13.08
Fat-free mass, kg	48.01 ± 9.11	54.74 ± 6.74*	41.29 ± 5.40
**Strength parameters**			
Relative hand grip	0.54 ± 0.14	0.59 ± 0.15	0.48 ± 0.12
Right hand grip, kg	39.05 ± 11.88	46.30 ± 11.97*	31.80 ± 6.17
Left hand grip, kg	35.40 ± 10.53	42.10 ± 9.86*	28.70 ± 6.09
Bench press 1RM, kg	31.92 ±15.54	43.88 ± 14.03*	21.15 ± 6.00
Leg press 1RM, kg	201.95 ± 79.66	261.11 ± 61.73*	148.70 ± 51.53
Biceps curl 1RM, kg	24.39 ± 9.04	31.05 ± 7.82*	18.40 ± 4.99
**Biochemical measures**			
Irisin, ng/mL	115.62 ± 72.41	85.23 ± 77.45*	142.96 ± 58.33
Glucose, mg/dL	91.61 ± 9.45	90.77 ± 11.33	92.44 ± 7.73
Cholesterol, mg/dL	179.61 ± 46.30	178.88 ± 53.21	180.33 ± 41.52
HDL, mg/dL	43.77 ± 9.58	38.77 ± 8.96*	48.77 ± 7.64
LDL, mg/dL	103.00 ± 41.73	105.12 ± 48.35	101.11 ± 37.79
VLDL, mg/dL	33.00 ± 19.55	34.25 ± 18.66	31.88 ± 21.88
Triglycerides, mg/dL	190.00 ± 115.69	210.44 ± 130.32	160.55 ± 102.56
**Medication**			
Infection duration, years	7.85 ± 7.10	6.90 ± 8.06	8.80 ± 6.28
HAART	6.05 ± 5.39	4.60 ± 5.03	7.50 ± 5.60

BMI: body mass index; HDL: high-density lipoprotein; LDL: low-density lipoprotein; VLDL: very-low-density lipoprotein; HAART: highly active antiretroviral therapy.

* Significantly different from women (p < 0.05).

### Clinical assessments

The present study was designed to quantify the circulating irisin concentration and determine possible correlations of irisin levels with fat mass, fat-free mass, BMI, and muscle strength in subjects with HIV. All testing and procedures were conducted between 2:00 – 5:30 p.m. Initially, self-reported demographics, and medical and HIV treatment history were obtained and confirmed by medical records. Targeted physical exams were obtained, including anthropometric measurements, maximal strength tests (one-repetition maximum, 1RM), and a hand grip strength test using a dynamometer. Prior to the physical evaluation, the participants reported to the laboratory between 7:00 – 10:00 a.m., following an overnight fast for blood sampling. Blood was collected from an antecubital vein for subsequent analysis of biochemical variables. HIV-1 RNA level and CD4+ lymphocyte count were obtained as part of a routine clinical care. All participants were encouraged to avoid smoking, alcohol, and caffeine consumption, as well as unusual physical activity before each test and blood sample collection.

### Determination of circulating irisin and other biochemical measurements

The participants reported to the laboratory between 08:00–10:00 a.m. after an overnight fast (12 hours), and blood samples (5 mL) were drawn from an antecubital vein into Vacutainer tubes (Becton Dickinson, Brazil). Briefly, samples were centrifuged at room temperature at 2,500 rpm for 15 min, and serum irisin concentrations were determined in duplicate using a commercial enzyme-linked immunosorbent assay (ELISA) kit (MyBioSource Inc., San Diego, CA, USA). Recently, Anastasilakis and cols. found that circulating irisin has a day-night variation with the lowest levels at 6:00 a.m. and the highest ones at 9:00 p.m. However, the blood collection was performed consistently at the same time of the day for individual participants (16). The minimum detectable level was 27.85 ng/mL. The intraassay and interassay coefficients of variation were 2.9–9.5% and 5.9–7.0%, respectively, and the sensitivity was 0.0093 pg/mL. Fasting plasma glucose, high-density lipoprotein (HDL), total cholesterol, and triglycerides were measured with colorimetric methods using a kit by Linco Research, Inc. (St. Charles, MO, USA) and by an automated analyzer (Human GmbH, Germany), while low-density lipoprotein (LDL) and very low-density lipoprotein (VLDL) were estimated by the Friedewald equation (17).

### Anthropometric and body composition analysis

Height was determined with a stadiometer, and body mass was recorded with a calibrated digital scale (Welmy^®^ W300, São Paulo, Brazil). Body composition was measured using bioelectrical impedance analysis (Maltron^®^ BF 906, São Paulo, Brazil) and absolute fat (kg), fat (%), absolute lean mass (kg), relative (%) lean mass, and BMI were obtained. The participants were advised to avoid alcohol, food or beverages containing stimulants, to ingest a minimum of 2 liters of water, and urinate up to 30 min before the test. On the assessment day, the participants’ earrings, chains and/or rings and shoes were removed. In order to perform the test, the participants were positioned in dorsal decubitus and electrodes were attached (left side) to their hands and feet.

### One-repetition maximum (1RM) muscle strength test

The participants performed two sessions of familiarization on bench press, 45° leg press, and biceps curl to establish the correct biomechanics of each movement. After the familiarization period, the participants performed a 1RM test and retest on two nonconsecutive days (minimum of 72 hours between tests). After 5 min of a light treadmill running, the participants performed eight repetitions of a specific warm-up with 50% of the estimated 1RM (according to the previous loads used by the participants in their familiarization sessions), followed by three repetitions with 70% of the estimated 1RM. Consecutive trials were performed for one repetition with progressively heavier loads. The 1RM was determined from three attempts, using 3- to 5-minute rest intervals between each attempt. The intraclass correlation coefficient was *r* = 0.96 for the tested exercises, thus confirming the test-retest reliability.

### Hand grip strength measurement

Isometric hand grip strength was determined by a mechanical hand dynamometer (Takei Digital Grip Strength Dynamometer, Model T.K.K., Takei, Scientific Instruments Co., Ltd., Niigata City, Japan). The participants stood up with both arms extended and their forearms in neutral rotation. The dynamometer grip was individually adjusted for each participant so that the interior stem of the dynamometer was positioned over the second phalanges of the index, middle, and annular fingers. The recovery time between measurements was 1 minute. The test was performed over three attempts in both hands. The best score of all three attempts was used as the value for each hand.

### Statistical analysis

Data are expressed as mean ± standard deviation (18). The Shapiro-Wilk test was applied to verify the normality of the distribution of the variables. Spearman’s test was used to verify the correlation of irisin levels with body composition parameters, BMI, strength variables, and HAART. An *r* value ≥ 0.5 determined a large correlation effect (19). To further investigate the potential predictors involved in irisin level changes, a multiple linear regression model was developed including the factors fat mass and fat-free mass adjusted for sex. The Mann-Whitney test was used for comparisons between the sexes. A *post hoc* power analysis for multiple linear regression (*R*^2^ increase) was calculated. Considering our estimated *R*^2^ of 0.57 for the entire regression model and the three predictors used, we estimated that a total sample size of 16 subjects was required to achieve a power level of 0.97 (Supplement 1). The *a priori* level of significance was p ≤ 0.05. The analyses were performed with the software SPSS, version 20.0 (SPSS Inc., Chicago, IL, USA), and G*Power 3.1.6 was used to calculate the statistical power (20,21).

The authors declare that the study design and procedures employed are in accordance with ethical standards and the Declaration of Helsinki. Each subject was fully informed about the risks associated with participation in the study and gave their written informed consent for participation. The study was approved by the Research Ethics Committee at Julio Muller University Hospital (protocol number: 673/09).

## RESULTS

**Figure 1:**
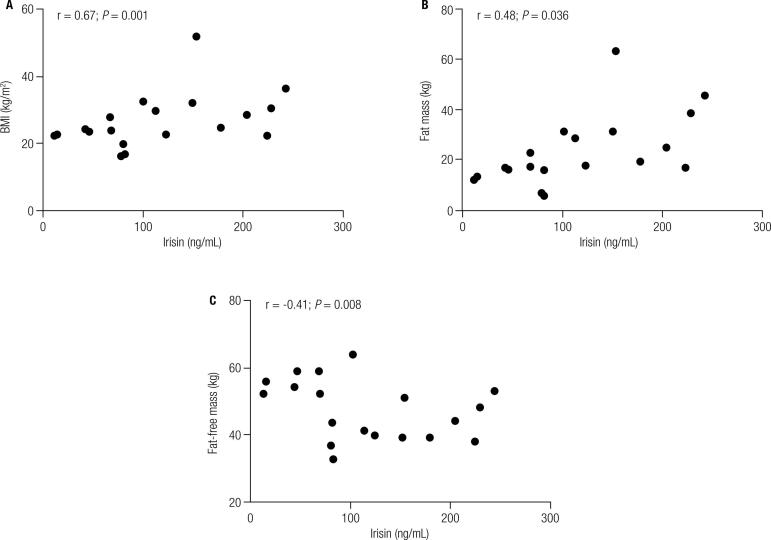
Correlation between irisin levels, parameters of obesity, and fat-free mass. (A) Correlation between body mass index (BMI) and irisin levels; (B) Correlation between fat mass and irisin levels; (C) Correlation between fat-free mass and irisin levels.

**Figure 2:**
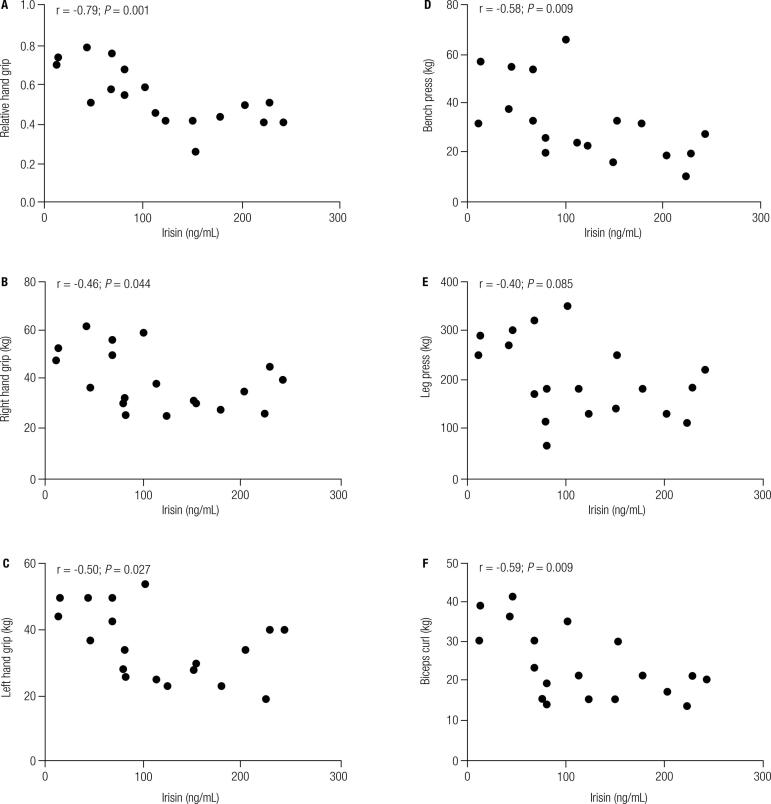
Correlation between irisin levels and strength variables. (A) Correlation between irisin levels and relative hand grip strength; (B) Correlation between irisin levels and right hand grip strength (kg); (C) Correlation between irisin levels and left hand grip strength (kg); (D) Correlation between irisin levels and bench press 1RM (kg); (E) Correlation between irisin levels and leg press 1RM (kg); (F) Correlation between irisin levels and biceps curl 1RM (kg).

Figure 1 presents the correlation of irisin levels with BMI, fat mass, and fat-free mass. Irisin levels correlated positively with two adiposity parameters: fat (*r* = 0.67; *p =* 0.001) and BMI (*r* = 0.48; *p* = 0.036). In contrast, there was an inverse correlation between irisin levels and fat-free mass (*r* = -0.41; *p* = 0.008) and each of the five strength parameters: relative hand grip (*r* = -0.79; *p* = 0.001), right hand grip (*r* = -0.46; *p* = 0.044), left hand grip (*r* = -0.50; *p* = 0.027), bench press 1RM (*r* = -0.58; *p* = 0.009), and biceps curl 1RM (*r* = -0.59; *p* = 0.009) (Figure 2). Although irisin levels showed an inverse correlation with leg press 1RM (*r* = -0.40; *p* = 0.085), this assessment was not statistically significant (Figure 2).

Differences between men and women were observed (Table 1). For the anthropometric measures, men exhibited higher fat-free mass when compared with women (*p* = 0.001). For the strength parameters, men exhibited a higher left and right hand grip strength (*p* = 0.002 and *p* = 0.004, respectively), bench press 1RM (*p* = 0.001), leg press 1RM (*p* = 0.001), and biceps curl 1RM (*p* = 0.001) when compared with women. For the biochemical markers, women presented higher HDL (*p* = 0.013) and irisin plasma levels as compared with men (*p* = 0.022). There were no differences in HIV infection duration between groups (*p* = 0.124). A numerical but not significant difference between men and women was observed regarding the duration of exposure to HAART (*p* = 0.062) (Table 1). In addition, the regression model predicting changes in irisin levels varied by 49% (*p* = 0.002) in regards to fat content and 25% to fat-free mass (*p* = 0.038), whereas sex was not statistically associated (*p* = 0.539) (Table 2).

**Table 2:** Independent effect of body composition on irisin lev

	B (95% confidence interval)	P value
Fat mass, kg	0.75 (1.70 – 6.03)	0.002*
Fat-free mass, kg	-0.66 (-10.51 – 0.34)	0.038*

Adjusted for sex (0 = men; 1 = women).

* Statistically significant (p < 0.05).

The results revealed weak positive correlations of HAART with BMI, percentage fat mass, fat mass, and percentage fat-free mass, and a negative and significant correlation with fat-free mass. In addition, there was a weak negative correlation of HAART with relative hand grip (*p* = 0.86), right hand grip (*p* = 0.22), and left hand grip (*p* = 0.16). The correlations between HAART and bench press (*p* = 0.02), leg press (*p* = 0.00), and biceps curl (*p* = 0.04) were negative and significant. The correlation between irisin levels and HAART was not statistically significant (*p* = 0.38) (Table 3).

**Table 3:** Correlation between highly active antiretroviral therapy (HAART), obesity parameters, and strength variables

	R	P
**Anthropometric measures**		
BMI, kg/m^2^	-0.74	0.75
Fat mass, %	0.03	0.87
Fat mass, kg	0.21	0.92
Fat-free mass, %	-0.03	0.85
Fat-free mass, kg	-0.49	0.02*
Strength parameters		
Relative hand grip	0.04	0.86
Right hand grip, kg	-0.28	0.22
Left hand grip, kg	-0.31	0.16
Bench press 1RM, kg	-0.52	0.02*
Leg press 1RM, kg	-0.65	0.00*
Biceps curl 1RM, kg	-0.46	0.04*
**Biochemical measures**		
Irisin, ng/mL	0.21	0.38

* Statistically significant (p < 0.05).

BMI: body mass index; HAART: highly active antiretroviral therapy.

## DISCUSSION

The aim of this study was to quantify the circulating irisin concentration in patients with HIV undergoing HAART and identify potential correlations between irisin levels and body composition and muscle strength. The initial hypothesis was confirmed, and the results revealed a positive correlation of irisin levels with body fat and negative correlation with fat-free mass and strength parameters in HIV-infected patients.

It is important to highlight the pathophysiological significance of the correlation between irisin levels, adiposity, fat-free mass, and strength variables. Our results contrast with the idea that the skeletal muscle is the main source of irisin secretion. Although the expression of the *FNDC5*/irisin gene in the muscle was not determined, irisin correlated positively with fat mass and negatively with fat-free mass. Intriguingly, we observed that 16 weeks of resistance training increased muscle strength and fat-free mass, and decreased fat mass, but did not change the circulating irisin levels in obese older women (22).

The source of circulating irisin in humans seems to be multicompartmental. Although the skeletal muscle is the tissue with the highest expression of *FNDC5*/irisin (2), other tissues, including the adipose tissue, are responsible for secreting irisin (6,7,23). Additionally, the expression of irisin is upregulated in individuals characterized as obese when compared with others who have a normal weight and are anorexic. Furthermore, irisin correlates positively with fat mass, BMI (11), fasting insulin, and HOMA-IR in women with gestational diabetes (12). Levels of irisin also correlate with reactive oxygen species, which play an important role in the development of insulin resistance and metabolic syndrome and are required for basal insulin signaling (24). Measurement of irisin from glycolytic versus oxidative muscle in response to acute exercise would also be an interesting assessment to compare different sources of irisin production (24).

Our findings raise two important hypotheses. Individuals with HIV present upregulated irisin receptors and/or irisin resistance at the receptor level, which may compromise the positive effects of irisin on glucose homeostasis and insulin sensitivity. Although insulin resistance was not analyzed in our study, we speculate that upregulation of the irisin receptor may positively influence the metabolic homeostasis in HIV-infected individuals. This is an interesting topic to be confirmed in future studies.

*FNDC5*/irisin expression has been found to be higher in obese patients with pre-diabetes and reduced by 40% in those with type 2 diabetes (23). In addition, circulating irisin levels have been shown to decrease following bariatric surgery in obese adults (25). Considering the possible benefits of BMI and body mass reduction, and the restoration of metabolic homeostasis, one would expect irisin levels to improve after surgery, but this was not the case (25). Moreover, circulating irisin levels appeared to be higher in women than men (25). This finding is in accordance with our cross-sectional study, in which HIV-infected women presented significantly higher baseline irisin levels as compared with men.

Pharmacological treatment may also influence irisin and HDL cholesterol levels. Recent data has revealed that lipid-lowering drugs affect circulating irisin levels in humans and PIs increase HDL cholesterol levels in women infected with HIV (26). In this sense, women presented longer infection and HAART durations when compared with men, although this finding was not significant, while a poor correlation between HAART and irisin levels was observed. Recently, Srinivasa and cols. investigated the effects of lifestyle modifications on circulating irisin and fibroblast growth factor 21 (FGF21) and their relationship with brown adipose tissue gene expression in dorsocervical subcutaneous fat. The authors demonstrated in individuals infected with HIV, a population prone to metabolic complications, that baseline FGF21 levels are increased when compared with a healthy cohort, and that FGF21 levels decrease relatively with lifestyle modifications. The effects of increased FGF21 may be to promote increased expression of brown adipose tissue genes in a unique ectopic adipose depot. In contrast, irisin levels are elevated in individuals infected with HIV but are not influenced by lifestyle modifications or associated with brown adipose tissue gene expression (13).

In previous studies that included pathological conditions, irisin correlated positively with BMI and fat mass and negatively with fat-free mass (25,27). In sedentary, overweight, and obese individuals, plasma irisin levels were positively associated with maximal contraction force and rate of force development after 12 weeks of resistance and endurance training (23). A decrease in irisin secretion contributes to muscle insulin resistance in high-fat diet mice (28). Thus, it is possible that the muscle/adipose irisin secretion ratio may vary according to the physiological condition of HIV-infected individuals. For example, the skeletal muscle and the adipose tissue are affected during HAART, which could modulate irisin levels. This has been confirmed by the negative correlation between HAART, fat-free mass, and strength parameters. These findings reinforce the need for further studies to elucidate whether irisin is involved in the HIV pathology and to verify the existence of irisin resistance and its correlation with metabolic disturbances present in HIV-infected subjects.

Of note, the real effect of irisin in humans remains unclear, as it has been shown that *FNDC5* is present in rodents and most primates, while humans present a mutation in the conserved start codon ATG to ATA. Thus, the results of irisin studies in humans should be interpreted with caution, and findings in mice should not be directly translated into humans (29). Moreover, serum irisin was measured by ELISA, which is based on polyclonal antibodies not previously tested for cross-reaction with serum proteins (30). However, it has been proposed that the variations in irisin concentrations found with different ELISA kits are consistent across independent studies with different cohorts of patients (31). The ELISA kit used in the present study has not been negatively tested in other studies and has acceptable levels of detection and reliability.

The present study has some limitations that should be considered, such as the small sample size and lack of a control group (individuals not infected with HIV or at an early stage of the disease), and a causal-effect relationship should not be interpreted from the results. Although our sample size was reduced, the sample power calculation revealed a moderate to strong effect size for all variables (data not shown). We initially tried to include a control group, but due to the patients’ lack of adherence and study dropout, it was impossible to include these data. The dropouts were mainly due to lack of benefits from participation in the study, as reported by the patients. Finally, we only assessed the plasma concentration of circulating irisin, while the expression of *FNDC5* in the skeletal muscle and adipose tissue was not determined.

In conclusion, irisin levels correlated positively with body fat and negatively with fat-free mass and strength parameters in HIV-infected subjects, which might suggest that the disturbances in adipose tissue and fat-free mass characteristic of the HIV disease could be responsible for the alterations in irisin levels. Future studies should determine the possible clinical relevance of these results in HIV patients.
